# Supplementary material for: Attosecond recorder of the polarization state of light
Source: Nat Commun. 2018 Feb 27;9:850. doi: 10.1038/s41467-018-03167-2 (PMC5829146; doi:10.1038/s41467-018-03167-2)
Supplement: Supplementary file 1 — Supplementary Material [file 41467_2018_3167_MOESM1_ESM.pdf]

Supplementary Material: Attosecond recorder of the polarization state of light

Á. Jiménez-Galán et al.

Supplementary Note 1: One photon ionization using pulses with time-dependent polarization

We write the partially polarized isolated attosecond pulse centered at time t_0 with field strength F_0, frequency Ω, envelope function $f_{\rm XUV}$, and degree of polarization $\mathcal{P} = \cos^2\gamma$, as

$$\mathbf{F}(t) = \mathrm{Re}\left\{F_0\, e^{-i\Omega(t-t_0)}\, f_{\rm XUV}\, \left[\boldsymbol{\sigma}_{\rm pol}(t)\cos\gamma + \boldsymbol{\sigma}_{\rm un}\sin\gamma\right]\right\}. \tag{1}$$

The polarized and unpolarized components, which have no correlation between them, are written, respectively, as

$$\begin{aligned}\boldsymbol{\sigma}_{\rm pol}(t) &= \frac{1}{\sqrt{1+|\epsilon(t)|^2}}\left[\hat{\mathbf{e}}_x + i\epsilon(t)\hat{\mathbf{e}}_y\right], \quad \text{and}\\ \boldsymbol{\sigma}_{\rm un} &= \frac{1}{\sqrt{2}}\left[\hat{\mathbf{e}}_x + i\, e^{i\phi}\hat{\mathbf{e}}_y\right] e^{i\beta},\end{aligned} \tag{2}$$

where $\epsilon(t)$ is the complex, time-dependent ellipticity. Its amplitude determines the usual ellipticity, while its phase determines the orientation of the polarization ellipse, that is, the phase between the x and y components. The phases ϕ and β are taken random and we assume the laboratory's x-axis is taken along the major axis of the ellipse. When the ellipticity changes as a function of time, it is useful to incorporate this time-dependence in an effective envelope function, which is different for the x and y components,

$$\boldsymbol{\sigma}_{\rm pol}(t) f_{\rm XUV}(t-t_0) = f^{(x)}_{\rm XUV}(t-t_0)\frac{\hat{\mathbf{e}}_x}{\sqrt{1+|\epsilon(t_0)|^2}} + i\,\epsilon(t_0) f^{(y)}_{\rm XUV}(t-t_0)\frac{\hat{\mathbf{e}}_y}{\sqrt{1+|\epsilon(t_0)|^2}}, \tag{3}$$

where the ellipticity is now a constant that is taken at the peak of the XUV field, and we have defined

$$\begin{aligned} f^{(x)}_{\rm XUV} &\equiv f_{\rm XUV}\frac{\sqrt{1+|\epsilon(t_0)|^2}}{\sqrt{1+|\epsilon(t)|^2}},\\ f^{(y)}_{\rm XUV} &\equiv f_{\rm XUV}\frac{\epsilon(t)\sqrt{1+|\epsilon(t_0)|^2}}{\epsilon(t_0)\sqrt{1+|\epsilon(t)|^2}}.\end{aligned} \tag{4}$$

Let us consider the one-photon ionization process. Since the polarized and unpolarized components are summed incoherently, we have two expressions for the transition amplitude. Provided the pulse ejects the photoelectron far from the core, the first order expression of the transition amplitude of a ground state $|\psi(\mathbf{r})\rangle$ to a continuum state represented by a plane wave $|\mathbf{k}\rangle$, may be written as

$$\begin{aligned} a_{\rm pol}(\mathbf{k}) &= -i\sqrt{2\pi}\,\frac{F_0}{\sqrt{1+|\epsilon(t_0)|^2}}\,\langle\mathbf{k}|\mathscr{F}_x d_x + i\,\epsilon(t_0)\,\mathscr{F}_y d_y|\psi(\mathbf{r})\rangle\,\cos\gamma,\\ a_{\rm un}(\mathbf{k}) &= -i\sqrt{2\pi}\,\frac{F_0}{\sqrt{2}}\,e^{i\beta}\,\mathscr{F}\langle\mathbf{k}|d_x + i\,e^{i\phi}\,d_y|\psi(\mathbf{r})\rangle\,\sin\gamma,\end{aligned} \tag{5}$$

where $\mathscr{F}_{x/y} = \int_{-\infty}^{\infty} dt\, f_{\rm XUV}^{(x/y)} e^{-i(t-t_0)\left(\Omega - k^2/2 - I_p\right)}$ denotes the Fourier transform of the field, with I_p being the ionization potential of the atom. For a hydrogenic system, we may write

$$\begin{aligned} d_x(\mathbf{k}) &\equiv \langle \mathbf{k}|d_x|\psi(\mathbf{r})\rangle = k_x g(k), \\ d_y(\mathbf{k}) &\equiv \langle \mathbf{k}|d_y|\psi(\mathbf{r})\rangle = k_y g(k), \end{aligned} \tag{6}$$

with $g(k)$ being an arbitrary well-behaved function of $k = \sqrt{k_x^2 + k_y^2}$. Furthermore, in the plane wave approximation, $g(k)$ is real. With this, the first order transition probability in the x direction reads

$$\begin{aligned} |a_{\rm tot}(k_x, k_y = 0)|^2 &= |a_{\rm pol}(k_x, k_y = 0)|^2 + |a_{\rm un}(k_x, k_y = 0)|^2 = \\ &= 2\pi|\mathscr{F}_x|^2|g(k_x)|^2 k_x^2 \left(\cos^2\gamma \frac{1}{1 + |\epsilon(t_0)|^2} + \frac{\sin^2\gamma}{2}\right), \end{aligned} \tag{7}$$

where we have assumed that the temporal profiles of the polarized and unpolarized components are essentially equivalent. Analogously for the y-direction,

$$|a_{\rm tot}(k_x = 0, k_y)|^2 = 2\pi|\mathscr{F}_y|^2|g(k_y)|^2 k_y^2 \left(\cos^2\gamma \frac{|\epsilon(t_0)|^2}{1 + |\epsilon(t_0)|^2} + \frac{\sin^2\gamma}{2}\right). \tag{8}$$

Thus, the ratio of the axes in the linear measurement of the polarization ellipse is

$$\frac{|a_{\rm tot}(k_x = 0, k_y = k)|^2}{|a_{\rm tot}(k_x = k, k_y = 0)|^2} = \frac{|\mathscr{F}_y|^2}{|\mathscr{F}_x|^2} \frac{2|\epsilon(t_0)|^2\cos^2\gamma + (1 + |\epsilon(t_0)|^2)\sin^2\gamma}{2\cos^2\gamma + (1 + |\epsilon(t_0)|^2)\sin^2\gamma}. \tag{9}$$

Even if the temporal profiles in the x and y directions are the same, the ellipticity and degree of polarization are intertwined in a one-photon measurement, and cannot be determined separately. Indeed, a perfectly polarized circular pulse ($\epsilon(t) = 1$, $\cos^2\gamma = 1$) will yield the same result as an unpolarized field ($\cos^2\gamma = 0$). To disentangle the ellipticity and degree of polarization, which will enable us to fully characterize the pulse, we must employ a second field.

Supplementary Note 2: Angular streak camera. Calculation of average signal and asymmetries

In our derivation we will consider an additional monochromatic infrared (IR) field linearly polarized along the x-direction,

$$\mathbf{F}_{\rm IR}(t) = F_{0,\rm IR}\sin(\omega t + \varphi_{\rm IR})\hat{\mathbf{e}}_x. \tag{10}$$

The expression of the transition amplitude of populating a state $|\mathbf{k}\rangle$ with kinetic momentum $\mathbf{k}$ at a moment T after the end of both the XUV pulse and IR-dressing field in the strong field approximation (SFA) [1–3] is

$$a(\mathbf{k}) = -i\int_{-\infty}^{T} dt \langle \mathbf{k} + \mathbf{A}_{\rm IR}(t)|\mathbf{F}(t)\cdot\mathbf{d}|\psi(\mathbf{r})\rangle e^{-i/2\int_t^T[\mathbf{k}+\mathbf{A}_{\rm IR}(\tau)]^2 d\tau} e^{-iI_p(T-t)}, \tag{11}$$

where $\mathbf{A}_{\rm IR}(t) = -\int dt \mathbf{F}_{\rm IR}(t)$ is the vector potential of the IR field. Let us again compute separately the transition amplitude of the polarized and unpolarized components. The calculations are almost standard for this type of problem. They take into account that i) the Volkov phase in the exponent is large and hence the integrand is rapidly oscillating, and ii) the attosecond pulse is ultrashort, which confines the integral to the vicinity of the peak t_0 of the attosecond pulse. Therefore, smoothly changing pre-exponential functions can be taken out of the integral at the peak t_0 of the XUV pulse. The only exception is made for the photo-ionization dipole, where we account for the streaking-field driven acceleration of the electron during photo-ionization (see below).

With the above discussion in mind, for the polarized component we simplify the integral as follows,

$$\begin{aligned} a_{\rm pol}(\mathbf{k}) = & -i\, F_0 \frac{\cos\gamma}{\sqrt{1+|\epsilon(t_0)|^2}} \int_{-\infty}^{T} dt\, e^{-i\Omega(t-t_0)} e^{-i/2\int_t^T [\mathbf{k}+\mathbf{A}_{\rm IR}(\tau)]^2 d\tau} e^{-iI_p(T-t)} \times \\ & \times \langle \mathbf{k}+\mathbf{A}_{\rm IR}(t)| f_{\rm XUV}^{(x)}\, d_x + i\,\epsilon(t_0)\, f_{\rm XUV}^{(y)}\, d_y |\psi(\mathbf{r})\rangle. \end{aligned} \tag{12}$$

Usually, one approximates the transition dipole as

$$d[\mathbf{k}+\mathbf{A}_{\rm IR}(t)] \approx d[\mathbf{k}+\mathbf{A}_{\rm IR}(t_0)]. \tag{13}$$

Here, we take into the account the fact that the electron momentum changes during the XUV pulse. To do this, we expand $\mathbf{k}(t)$ around $\mathbf{k}(t_0)=\mathbf{k}_0$,

$$\mathbf{k}_0 = \mathbf{k} + \mathbf{A}_{\rm IR}(t_0), \tag{14}$$

so that

$$\mathbf{k} + \mathbf{A}_{\rm IR}(\tau) = \mathbf{k}_0 + \Delta\mathbf{k}(\tau), \tag{15}$$

where $\Delta\mathbf{k}(\tau) \equiv \mathbf{A}_{\rm IR}(\tau) - \mathbf{A}(t_0)$. Now we expand this difference to first order,

$$\Delta\mathbf{k}(\tau) \approx \Delta\mathbf{k}(t_0) + \left.\frac{\partial \Delta\mathbf{k}(\tau)}{\partial\tau}\right|_{t_0} (\tau - t_0) = \left.\frac{\partial \mathbf{A}_{\rm IR}(\tau)}{\partial\tau}\right|_{t_0} (\tau - t_0) = -(\tau - t_0)\mathbf{F}_{\rm IR}(t_0). \tag{16}$$

In this way, the dipole matrix elements may be approximated as

$$\begin{aligned} d_x(\mathbf{k}+\mathbf{A}_{\rm IR}(t)) &\approx d_{0x} - d'_{0x}(t-t_0)F_{\rm IR}(t_0), \\ d_y(\mathbf{k}+\mathbf{A}_{\rm IR}(t)) &\approx d_{0y} - d'_{0y}(t-t_0)F_{\rm IR}(t_0), \end{aligned} \tag{17}$$

where $d_{0x/y} = d_{x/y}(\mathbf{k}_0)$ is the usual zero-order correction and $d'_{0x/y} = \frac{\partial \langle \mathbf{k}(t)|d_{x/y}|\Psi_g(\mathbf{r})\rangle}{\partial k_x}|_{\mathbf{k}(t)=\mathbf{k}_0}$ is the first order correction, that is, the derivative of the dipole moment with respect to the streaked momentum in the direction parallel to the laser polarization axis, and evaluated at k_0. Inserting Eq. (17) in Eq. (12) we obtain

$$a_{\rm pol}(\mathbf{k}) = -i \frac{\cos\gamma}{\sqrt{1+|\epsilon(t_0)|^2}} \left[a_{{\rm pol},x} + i\,\epsilon(t_0)\, a_{{\rm pol},y}\right], \tag{18}$$

where

$$\begin{aligned} a_{\mathrm{pol},x} &= \mathcal{F}_x\, d_{0x} - i\, F_{\mathrm{IR}}(t_0)\, \mathcal{F}'_x\, d'_{0x}, \\ a_{\mathrm{pol},y} &= \mathcal{F}_y\, d_{0y} - i\, F_{\mathrm{IR}}(t_0)\, \mathcal{F}'_y\, d'_{0y}, \end{aligned} \tag{19}$$

and we have defined

$$\begin{aligned} \mathcal{F}_{x/y} &\equiv \int_{-\infty}^{T} dt\, F_0\, f_{\mathrm{XUV}}^{(x/y)}\, e^{-i\Omega(t-t_0)} e^{-i/2\int_t^T [\mathbf{k}+\mathbf{A}_{\mathrm{IR}}(\tau)]^2 d\tau} e^{-iI_p(T-t)}, \\ \mathcal{F}'_{x/y} &= \frac{\partial \mathcal{F}_{x/y}}{\partial \Omega}. \end{aligned} \tag{20}$$

These represent the usual streak-camera spectrograms for the x and y components [4]. The transition probability for the polarized component can thus be written as

$$|a_{\mathrm{pol}}(\mathbf{k})|^2 = \frac{\cos^2\gamma}{1+\epsilon(t_0)^2}\left[|a_{\mathrm{pol},x}|^2 + \epsilon(t_0)^2\, |a_{\mathrm{pol},y}|^2 + i\,\epsilon\left(a^*_{\mathrm{pol},x}\, a_{\mathrm{pol},y} - a_{\mathrm{pol},x}\, a^*_{\mathrm{pol},y}\right)\right]. \tag{21}$$

As usual for the attosecond streak camera, we can neglect terms of the order $\mathrm{O}(F_{\mathrm{IR}})$ in the first two terms, so that

$$|a_{\mathrm{pol},x/y}|^2 = |d_{0x/y}|^2 |\mathcal{F}_{x/y}|^2. \tag{22}$$

For the cross terms, however, we have to keep the linear order terms in the IR field, since the zero-order terms in F_{IR} cancel. Indeed, from Eq. (19) we have

$$a^*_{\mathrm{pol},x}\, a_{\mathrm{pol},y} = \mathcal{F}^*_x \mathcal{F}_y\, d^*_{0x}\, d_{0y} - i\, F_{\mathrm{IR}}(t_0)\left(\mathcal{F}^*_x\, \mathcal{F}'_y\, d^*_{0x}\, d'_{0y} - \mathcal{F}'^*_x\, \mathcal{F}_y\, d_{0y}\, d'^*_{0x}\right), \tag{23}$$

and

$$a_{\mathrm{pol},x}\, a^*_{\mathrm{pol},y} = \mathcal{F}_x \mathcal{F}^*_y\, d_{0x}\, d^*_{0y} - i\, F_{\mathrm{IR}}(t_0)\left(\mathcal{F}'_x\, \mathcal{F}^*_y\, d'_{0x}\, d^*_{0y} - \mathcal{F}_x\, \mathcal{F}'^*_y\, d_{0x}\, d'^*_{0y}\right). \tag{24}$$

If the envelopes of the x and y components of the attosecond XUV pulse are similar, we may introduce the average streaked image $\mathcal{F}$ and write

$$\begin{aligned} \mathcal{F}_x &\approx \mathcal{F} + \Delta\mathcal{F}_x, \quad \text{and} \\ \mathcal{F}_y &\approx \mathcal{F} + \Delta\mathcal{F}_y, \end{aligned} \tag{25}$$

where the second terms in the RHS of the expressions above are a small correction. Since already the terms linear in $F_{\mathrm{IR}}(t_0)$ of Eqs. (23) and (24) are small, using Eq. (25) we may write

$$\mathcal{F}^*_x \mathcal{F}'_y \approx \mathcal{F}'_x \mathcal{F}^*_y \simeq \mathcal{F}^* \mathcal{F}', \quad \text{and} \quad \mathcal{F}'^*_x \mathcal{F}_y \approx \mathcal{F}_x \mathcal{F}'^*_y \simeq \mathcal{F}'^* \mathcal{F}. \tag{26}$$

With this, and using Eq. (6), we may express the polarized component of the transition probability in an appealing form

$$|a_{\mathrm{pol}}(\mathbf{k})|^2 = \frac{g(k_0)^2 \cos^2\gamma}{1+\epsilon(t_0)^2}\left[\left(|\mathcal{F}_x|^2 k^2_{0x} + |\mathcal{F}_y|^2 \epsilon(t_0)^2\, k^2_{0y}\right) - \epsilon(t_0)\, k_{0y}\, F_{\mathrm{IR}}(t_0) \frac{\partial |\mathcal{F}|^2}{\partial\Omega}\right]. \tag{27}$$

Following analogous steps, we arrive to the expression of the unpolarized component,

$$|a_{\rm un}(\mathbf{k})|^2 = \frac{\sin^2\gamma}{2}\, g(k_0)^2 \left(|\mathcal{F}_x|^2 k_{0x}^2 + |\mathcal{F}_y|^2 k_{0y}^2\right), \tag{28}$$

whose main difference with the polarized component is the absence of cross terms. The temporal profiles we assume to coincide with that of the polarized component on the basis that the attosecond XUV light comes out from the high harmonic generation process, where, due to depolarization of the harmonics [5], it is not perfectly polarized, but shares a common envelope for both the polarized and unpolarized components. We note that the above approximation may not be justified in the case where $|\mathcal{F}_x|^2$ is very different from $|\mathcal{F}_y|^2$, which happens when the ellipticity has a very complicated time-dependence; however, it holds for all the cases we have investigated in this work.

The measured transition probability is then the incoherent sum of the polarized and unpolarized components,

$$|a(\mathbf{k})|^2 = |a_{\rm pol}(\mathbf{k})|^2 + |a_{\rm un}(\mathbf{k})|^2. \tag{29}$$

According to Eq.(3) of the main manuscript, the average signal is defined as

$$S(k_x, k_y) = (|a(k_x, k_y)|^2 + |a(k_x, -k_y)|^2)/2. \tag{30}$$

With this and the expressions for the polarized and unpolarized transition amplitudes, we may write explicitly the average signal as

$$S(k_x, k_y) = g(k_0)^2 \left\{ |\mathcal{F}_x|^2 k_{0x}^2 \left(\frac{\cos^2\gamma}{1+\epsilon(t_0)^2} + \frac{\sin^2\gamma}{2} \right) + |\mathcal{F}_y|^2 k_{0y}^2 \left(\frac{\epsilon(t_0)^2\cos^2\gamma}{1+\epsilon(t_0)^2} + \frac{\sin^2\gamma}{2} \right) \right\}. \tag{31}$$

Measuring the average signal along the direction parallel to the IR polarization axis (along $\hat{\mathbf{e}}_x$ in this case), yields the same expression as that used in the attosecond streak camera [4],

$$S(k_x, k_y) \propto |\mathcal{F}_x|^2\, \sigma_{\rm cs}(k_0), \tag{32}$$

where $\sigma_{\rm cs}(k_0) = k_0^2\, g(k_0)^2$ is proportional to the cross section. From the expression above, one can obtain the streaking spectrogram $\mathcal{F}_x$ by varying the XUV-IR time delay and using a FROG-CRAB retrieval algorithm [6]. Analogously, from the experiment in which the IR is oriented perpendicular to the major axis of the XUV ellipse (along $\hat{\mathbf{e}}_y$ in this case), one obtains the spectrogram $\mathcal{F}_y$. By comparing the streaking traces in both $\hat{\mathbf{e}}_x$ and $\hat{\mathbf{e}}_y$ directions, one obtains the relative time center of the two profiles $\mathcal{F}_x$ and $\mathcal{F}_y$.

To obtain the explicit formula for the asymmetry, we begin from the definition given in Eq.(3) of the main manuscript:

$$A(k_x, k_y) = (|a(k_x, k_y)|^2 - |a(k_x, -k_y)|^2)/S(k_x, k_y), \tag{33}$$

and use the expressions for the transition amplitude of the polarized and unpolarized components. For the present configuration, in which the IR is oriented along $\hat{\mathbf{e}}_x$ ($\hat{\mathbf{e}}_x$ coinciding with the major axis of the XUV ellipse), we obtain,

$$A_1(k_x, k_y) = -2\, g(k_0)^2\, \epsilon\, k_{0y}\, F_{\text{IR}}(t_0)\, \frac{\partial|\mathcal{F}|^2}{\partial\Omega} \frac{\cos^2\gamma}{1+\epsilon(t_0)^2} \frac{1}{S_1(k_x, k_y)}. \tag{34}$$

If we measure the asymmetry along $\hat{\mathbf{e}}_y$, that is, in the direction perpendicular to the IR polarization axis, and take the average spectrogram $\mathcal{F} \approx \mathcal{F}_y$, the above expression simplifies to the one given in Eq.(4) of the main manuscript by realizing that

$$\frac{\partial|\mathcal{F}|^2}{\partial\Omega} = \frac{1}{\frac{\epsilon(t_0)^2\,\cos^2\gamma}{1+\epsilon(t_0)^2} + \frac{\sin^2\gamma}{2}} \frac{\partial[S_1(k_y, t_0)/\sigma_{\text{cs}}(k_0)]}{\partial\Omega}, \tag{35}$$

In a similar way, Eq.(7) of the main manuscript can be derived by considering the IR polarized along $\hat{\mathbf{e}}_y$ and measuring the signal along $\hat{\mathbf{e}}_x$, that is, $k_{0x} = k_0$.

Supplementary Note 3: Comparison with full solution of the TDSE for hydrogen

To test our theoretical predictions, we performed several numerical experiments. We solved in a virtually exact way the TDSE for the ionization of the hydrogen atom by a partially polarized, elliptical XUV pulse in the presence of a linearly polarized IR laser field. We simulated the signal of a partially polarized pulse as

$$\text{Signal} = I_{\text{pol}} \cos^2\gamma + \frac{I_{\text{perp}} + I_{\text{par}}}{2} \sin^2\gamma. \tag{36}$$

The first term in the RHS is the polarized component while the second term is the unpolarized component. In particular, I_{pol} is the spectrum obtained from a simulation with a perfectly polarized XUV pulse plus a linearly polarized IR, I_{perp} is the spectrum obtained from a simulation with a linear XUV pulse plus a linear IR field polarized in the perpendicular direction with respect to the XUV polarization axis, and I_{par} is the spectrum obtained from a simulation with a linear XUV pulse plus a linear IR field polarized along the same direction as the XUV. By doing so, we fulfill the two necessary and sufficient conditions, that is i) polarized and unpolarized fields are uncorrelated and ii) x and y components of the unpolarized light are uncorrelated.

The inputted polarized components of the vector potential (shown as purple and orange lines in Fig.2 of the main manuscript) are:

$$\begin{aligned} A_x^{\text{inp}}(t) &= \frac{1}{1+\epsilon(t)^2} F_X \exp\{-2\log(2)[t-t_0]^2/\tau^2\}\, \cos[\Omega(t-t_0)+\phi_x(t)], \quad \text{and} \\ A_y^{\text{inp}}(t) &= \frac{\epsilon(t)}{1+\epsilon(t)^2} F_X\, \exp\{-2\log(2)[t-t_0]^2/\tau^2\}\, \sin[\Omega(t-t_0)+\phi_y(t)], \end{aligned} \tag{37}$$

where $F_X = 0.001$ a.u., $\Omega = 1.5$ a.u., $\tau = 10.35$ a.u., and the time-dependent ellipticity was $\epsilon(t) = 0.95 + 0.65\mathrm{Erf}\,[0.15(t - t_0 - 1.5)]$ for the pulse in Fig.2a of the main manuscript and $\epsilon(t) = 0.7 + 0.2\mathrm{Erf}\,[0.2(t - t_0)]$ for Fig.2b of the main manuscript. It is clear that the resulting envelope of the pulses above is not Gaussian nor is it the same in the x and y directions. The IR laser field had also a Gaussian shape and field strength of $F_{\mathrm{IR}} = 0.01$ a.u., duration $\tau_{\mathrm{IR}} = 300$ a.u. and frequency $\omega_{\mathrm{IR}} = 0.0569$ a.u.

For the reconstruction of the polarized components of the XUV pulse from the spectrograms (shown as black lines and dots in Fig.2 of the main manuscript), we assumed Gaussian envelopes and accounted for the two time delays in the spectrograms, one due to the Wigner delay and another due to the Coulomb-laser coupling (free-free) delay [7]. In particular,

$$\begin{aligned} A_x^{\mathrm{rec}}(t) &= F_X \exp\{-2\log(2)[t - (t_{0x} + \tau_W + \tau_{CC})]^2/\tau_x^2\} \cos[\Omega(t - t_0) + \phi_x(t)], \quad \text{and} \\ A_y^{\mathrm{rec}}(t) &= F_X\, \epsilon(t_0) \exp\{-2\log(2)[t - (t_{0y} + \tau_W + \tau_{CC})]^2/\tau_y^2\} \sin[\Omega(t - t_0) + \phi_y(t)], \end{aligned} \tag{38}$$

where $t_{0x/y}$ is the time center of the temporal profiles in the x/y direction which is obtained from the streaked trace, t_0 is the average of these two times, τ_Ω is the photoionization time delay, which is composed of the Wigner and coulomb-laser-coupling time delays. The duration of the temporal profiles $\tau_{x/y}$ is obtained from the streaking spectrogram along with the chirp $\phi_{x/y}(t)$, or from the measurement of the photoelectron distribution along the direction perpendicular to the IR polarization axis at one time delay, if no chirp is present. In order to reduce computational time, the IR pulse used was shorter than those generally used in experiments; thus, to minimize the influence of the short IR pulse duration in the temporal profile of the XUV field, we assumed no chirp and measured the photoelectron signal in the direction perpendicular to the IR polarization axis. By fitting this profile to a Gaussian envelope, we obtained the spectral widths of the distributions, from which the temporal duration τ_x and τ_y were obtained. Finally, $\epsilon(t_0)$ is the ellipticity at the center of the pulse, obtained from the asymmetry curve as discussed in the main text. Ellipticity at all other times is now obtained using the already reconstructed x, y components from the usual streak-camera traces.

Additionally to the reconstructions in the main manuscript, we also reconstructed the pulse in Fig.2a of the main manuscript for a degree of polarization of 0.85. The orientation of the major axis of the XUV ellipse had a 15 degrees offset with respect to the x-axis. Nonetheless, to test the robustness of our method with respect to a few degrees offset, we have oriented the IR polarization axis parallel and perpendicular to the x-axis. The observables extracted for this pulse are shown in Supplementary Figure 1. The retrieved durations and time centers of the temporal profiles in the x and y directions are similar to those retrieved in the case of Fig.2a of the main manuscript (perfect polarization). Specifically, in the case of the 15% mixture of unpolarized light (Supplementary Figure 1), the peak of the y-component is retarded with respect to the x-component by $t_{0,y} - t_{0,x} = 60$ as, while in the case of perfect polarization that difference was $t_{0,y} - t_{0,x} = 70.7$ as.

The duration of the temporal profile extracted for the x-component was the same for both partially polarized and completely polarized light, whilst that for the y-component was $\tau_y = 229$ as for the partially polarized pulse and $\tau = 225$ as for the perfectly polarized pulse. The ellipticity at the pulse center was $\epsilon(t_0) = 0.793$ for the partially polarized and $\epsilon(t_0) = 0.784$ for the perfectly polarized pulse. The slight differences between these parameters are due to the small discrepancy between the temporal profiles of the polarized and unpolarized components, which is a consequence of having a complicated time-varying ellipticity. Finally, the degree of polarization extracted for the partially polarized light was $\cos^2\gamma = 0.838$, in excellent agreement with the value of $\cos^2\gamma = 0.85$ that was used.

Besides the reconstructions of pulses with time-dependent ellipticity shown in the main manuscript, we additionally performed four simulations for pulses with constant value of the ellipticity and longer duration. In all of them, $F_X = 0.001$ a.u., $\tau = 14$ a.u. and $\Omega = 1.5$ a.u and the IR laser field had a field strength of $F_{\text{IR}} = 0.00569$ a.u., duration $\tau_{\text{IR}} = 300$ a.u. and frequency $\omega_{\text{IR}} = 0.0569$ a.u. What we changed was the degree of polarization and the ellipticity of the XUV pulse. In particular, we simulated the following polarization state $(\epsilon, \cos^2\gamma)$ combinations: $(\epsilon = 0.6, \cos^2\gamma = 0.75)$, $(\epsilon = 0.6, \cos^2\gamma = 1$, that is, totally polarized), $(\epsilon = 0.9, \cos^2\gamma = 0.85)$ and $(\epsilon, \cos^2\gamma = 0$, that is, unpolarized). The time delay between the XUV pulse and IR field was chosen so that the maximum of the XUV pulse coincided with the minimum of the IR vector potential, where the model predicts the photoelectron asymmetry should be larger.

From the streaking spectrum, we extracted the positive and negative momentum distributions in the direction perpendicular to the laser polarization axis, for both laser orientations (parallel and perpendicular to the major axis of the XUV ellipse). To account for the experimental resolution, we integrated over an angular range of 11 degrees. The extracted photoelectron asymmetries and the fitted curve are shown in Supplementary Figure 2. The values of ϵ and γ extracted from the fit are reported in Supplementary Table 1. The difference with the inputted values was never higher than 2.5%.

Supplementary Note 4: Effects of CEP-noise in the reconstruction

We have tested the robustness of our technique by considering the effect of typical CEP-noise on the resulting averaged measurements. Specifically, we have performed the reconstruction for the same pulse as shown in Fig.2b of the manuscript, but now taking into account CEP jitter of 0.1 rad, typical in attosecond pump-probe experiments. To this end, we have performed five calculations for different CEP of the IR field: -0.1, -0.05, 0, 0.05 and 0.1 rad, and summed the photo-electron spectra incoherently, thus mimicking the experimental jitter. We have applied the same procedure as for the case of zero jitter, that is we have used the asymmetry in the spectra, now extracted from the jitter-averaged spectra. The results are shown

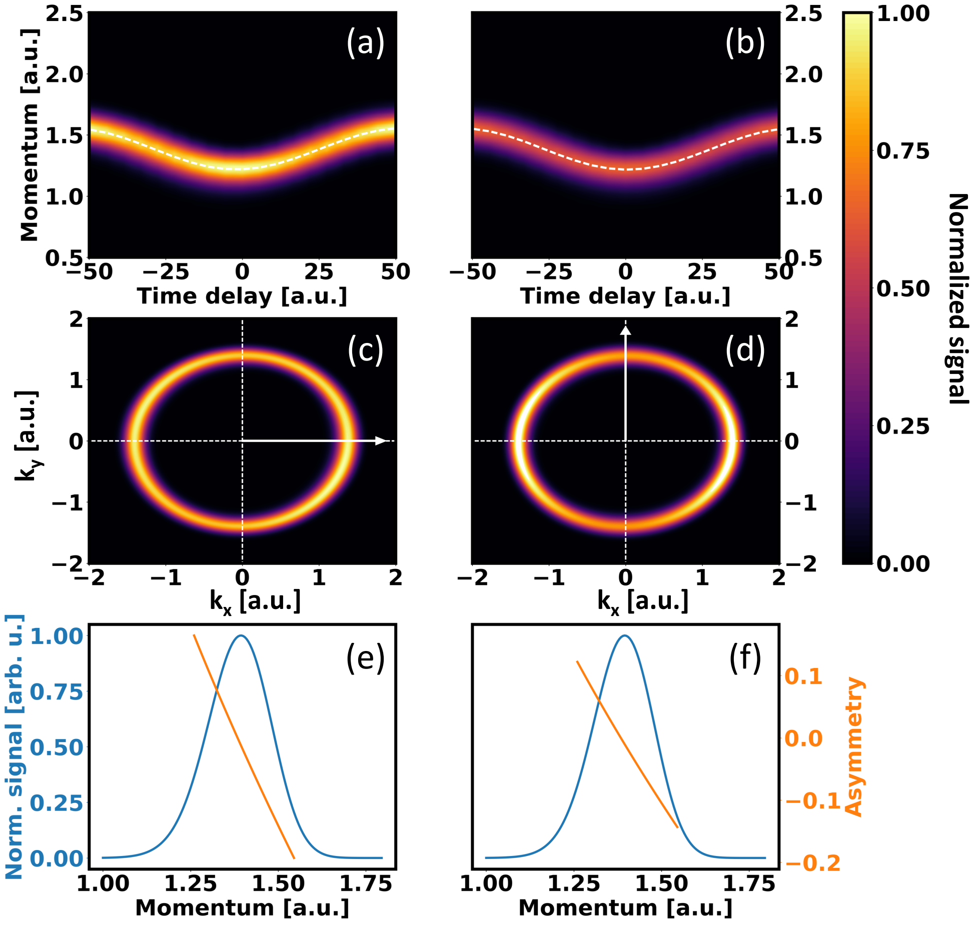

Supplementary Figure 1: **Measured observables relevant for the reconstruction**. Observables obtained from the photoionization of the hydrogen atom by the pulse in Fig.2a of the main manuscript with an admixture of 15% unpolarized light, in the presence of the IR field described in the main manuscript. The major axis of the XUV ellipse is tilted by around 15 degrees with respect to the x-axis and the IR field is oriented along the x-axis in the left columns and along the y-axis in the right columns. Panels (a,b): streaking traces measured along the x direction (a) and y direction (b). Panels (c,d): angularly-resolved photoelectron distribution streaked by an IR field polarized along the x direction (c) and y direction (d). Panels (e,f): temporal profile of the pulse (blue line) and asymmetry between the two distributions measured perpendicular to the IR polarization axis (orange lines) for IR polarized along the x direction (e) and y direction (f).

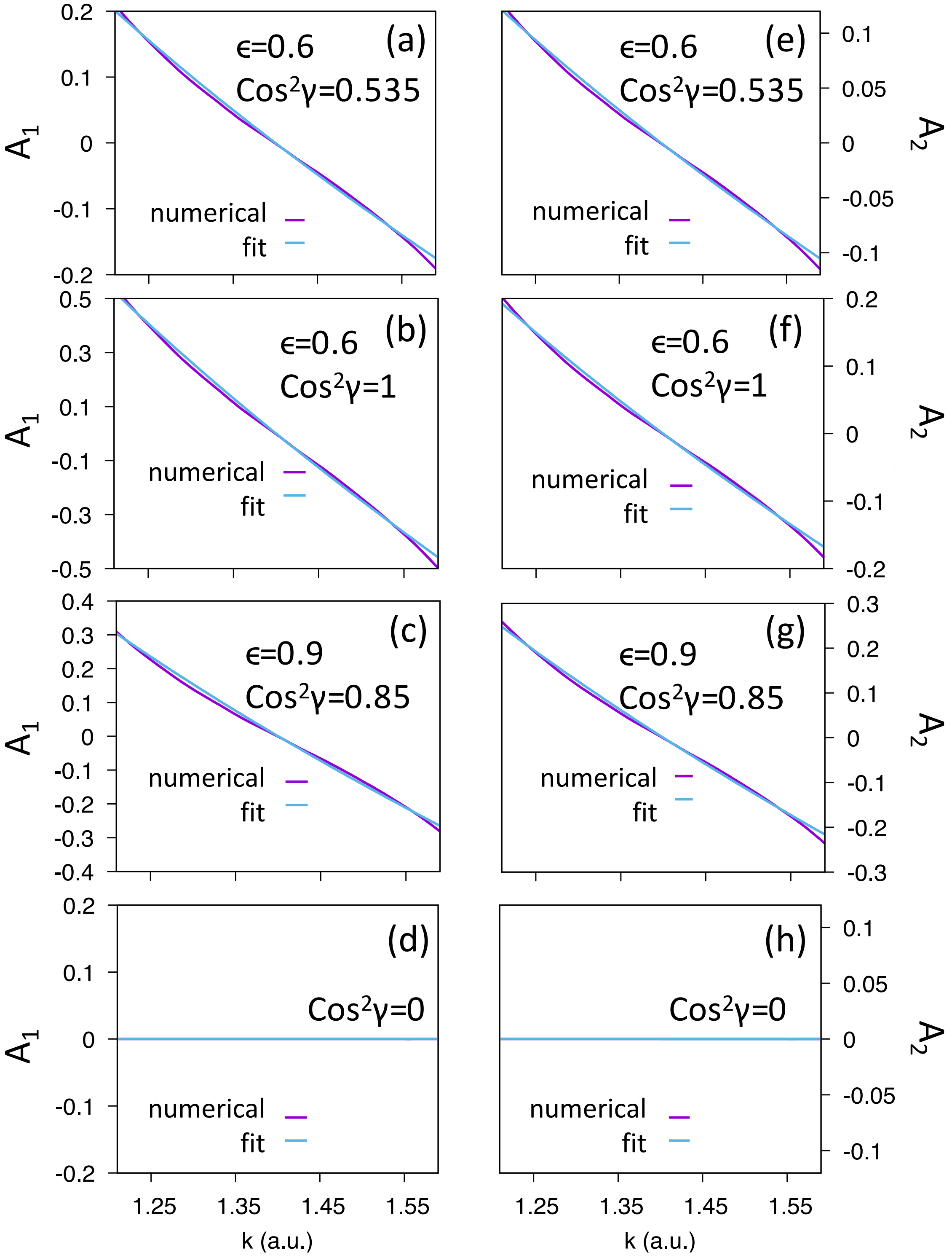

Supplementary Figure 2: **Fitting of photoelectron asymmetries**. Photoelectron asymmetries extracted from the spectrum (magenta line) for parallel (panels a-d) and perpendicular (panels e-h) IR orientations, and its fit with the corresponding model equations (blue line), for pulses with time-independent ellipticity.

	Theoretical	Retrieved from spectrum
ϵ	0.6	0.596 ± 0.004
$\cos^2\gamma$	0.535	0.521 ± 0.002
ϵ	0.6	0.595 ± 0.003
$\cos^2\gamma$	1	0.974 ± 0.003
ϵ	0.9	0.885 ± 0.005
$\cos^2\gamma$	0.85	0.836 ± 0.004
ϵ	–	–
$\cos^2\gamma$	0	$4 \cdot 10^{-11} \pm 1 \cdot 10^{-8}$

Supplementary Table 1: **Reconstructed values of ϵ and $\cos^2\gamma$.** Comparison between the theoretically inputted values of the XUV ellipticity ϵ and degree of polarization $\cos^2\gamma$, and those retrieved from the spectrum, for pulses with time-independent ellipticity.

in Supplementary Figure 3, together with the fit to our analytical formula, which is indistinguishable from the simulations. The extracted parameters are summarized in Supplementary Table 2, demonstrating robustness of our method.

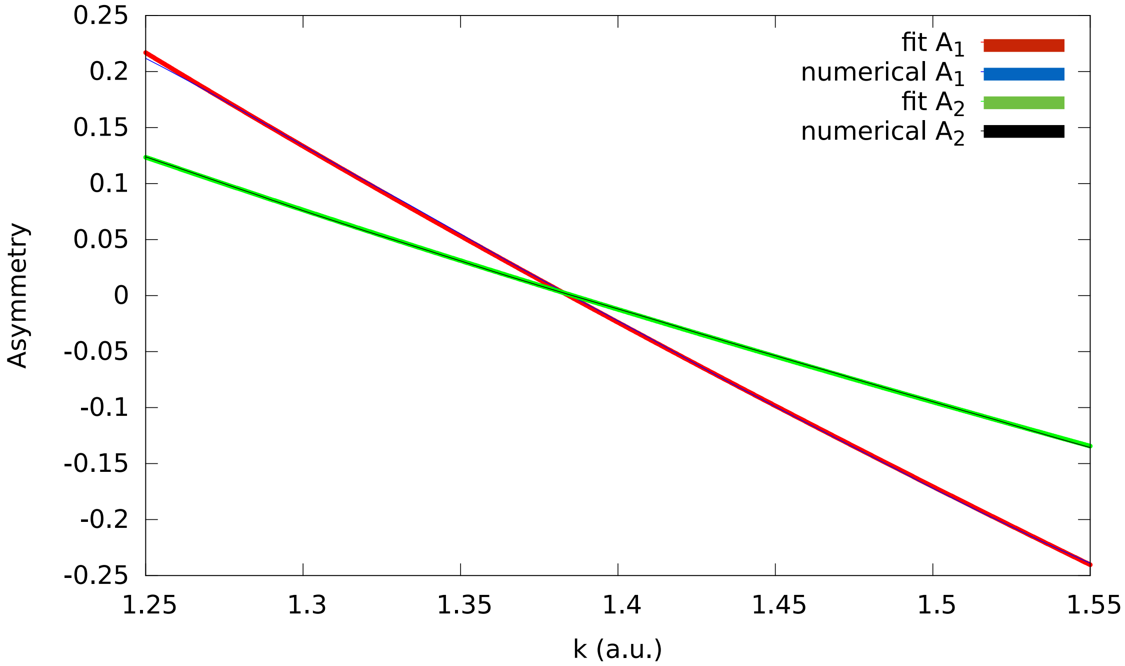

Supplementary Figure 3: **Asymmetries measured with CEP instability**. Asymmetries A_1 and A_2 (as defined in the manuscript) for the pulse in Fig.2b of the manuscript. The asymmetries are obtained for the case when there is an admixture of 15% of unpolarized light. The spectra are averaged over 0.1 rad CEP jitter. The extracted values of the ellipticity and degree of polarization are given in Supplementary Table 2 and compared to the case of perfect CEP stability.

	Theoretical	Reconstructed (no jitter)	Reconstructed (jitter)
$\epsilon(t_0)$	0.7	0.691	0.689
$\cos^2\gamma$	0.85	0.826	0.826
$\epsilon(t_0)$	0.7	0.686	0.682
$\cos^2\gamma$	1	0.99	0.99

Supplementary Table 2: **Polarization parameters reconstructed with CEP instability**. Comparison between the inputted $\epsilon(t_0)$ and $\cos^2\gamma$ values (left column) and the reconstructed values assuming perfect CEP stability (central column) and CEP jitter of 0.1 rad (right column). The pulse considered has the same time-dependent polarization state as that in Fig.2b of the main manuscript.

We note that one strong point of this method is its redundancy. The asymmetry depends on both the XUV-IR time delay and the angle; and the analytical formula can be easily derived for arbitrary time delay and arbitrary angle. If the experiment is fully angularly resolved (for example, when using a COLTRIMS apparatus), one can extract the asymmetry at many XUV-IR time delays and along many angles. This will further reduce the impact of experimental uncertainties such as the CEP jitter.

Supplementary Note 5: Arbitrary phase between x and y components of the polarized part

Time-independent phase

Consider first the simple case of arbitrary, but time-independent phase shift between the x and y components:

$$\begin{aligned} F_x(t) &\propto \cos\left[\omega_{\text{XUV}}(t-t_0)\right], \\ F_y(t) &\propto \epsilon\sin\left[\omega_{\text{XUV}}(t-t_0)+\varphi_0\right]. \end{aligned} \tag{39}$$

From the linear measurement, we can find the major and minor axes of the time-averaged polarization ellipse. They will be rotated by the angle α relative to the x and y axes:

$$\tan 2\alpha = \frac{2\epsilon\sin\varphi_0}{1-\epsilon^2}, \tag{40}$$

where ϵ is the ratio of the x and y components in the original frame. It is easy to check that, once we rotate our axes by α, the field components along the rotated x' and y' axes will have the $\pi/2$ phase-shift. Now we can apply our technique as before and characterize the pulse in the rotated frame (x',y'). Of course, the true value of the ellipticity ϵ' now differs,

$$\epsilon' = \frac{\epsilon\cos\alpha\cos\varphi_0}{\epsilon\sin\varphi_0\sin\alpha+\cos\alpha}, \tag{41}$$

while the phase ϕ is

$$\tan\phi = \frac{\epsilon\cos\varphi_0\sin\alpha}{\epsilon\sin\varphi_0\sin\alpha + \cos\alpha}. \tag{42}$$

Importantly, we will also already know the degree of polarization, provided by our technique.

As a test case with time-dependent polarization, we used the pulse defined by

$$\begin{aligned} F_x &= \frac{f_{\mathrm{XUV}}(t - t_{0\mathrm{XUV}})}{\sqrt{1+\epsilon(t)^2}}\cos[\omega_{\mathrm{XUV}}(t - t_{0\mathrm{XUV}})], \\ F_y &= \frac{\epsilon(t)\, f_{\mathrm{XUV}}(t - t_{0\mathrm{XUV}})}{\sqrt{1+\epsilon(t)^2}}\sin[\omega_{\mathrm{XUV}}(t - t_{0\mathrm{XUV}}) + \varphi_0], \end{aligned} \tag{43}$$

where $\epsilon(t) = 0.9 + 0.2\ \mathrm{Erf}[0.2(t - t_{0\mathrm{XUV}})]$ and φ_0 was randomly chosen between 0 and $\pi/2$, with the value $0.22(\pi/2)$.

First, we performed the linear measurement, see Supplementary Figure 4a. We clearly see the tilt in the polarization ellipse. Hence, the x and y components of the pulse are not shifted by $\pi/2$. We then rotated the reference frame so that the major axis in the linear measurement coincides with the x axis (and the minor axis coincides with the y axis). This is shown in Supplementary Figure 4b. The angle of rotation found from the linear measurement was $\alpha = 36.2°$. It coincides with that predicted by Eq. 40. In this rotated frame the x' and y' components of the field are shifted by $\pi/2$, and we apply our method. To obtain the reconstructed pulse in the original frame, we just need to rotate it back by angle α. The comparison between the pulse in Eq. (43) and the reconstructed pulse in the original reference frame (x,y) is shown in Supplementary Figure 4c.

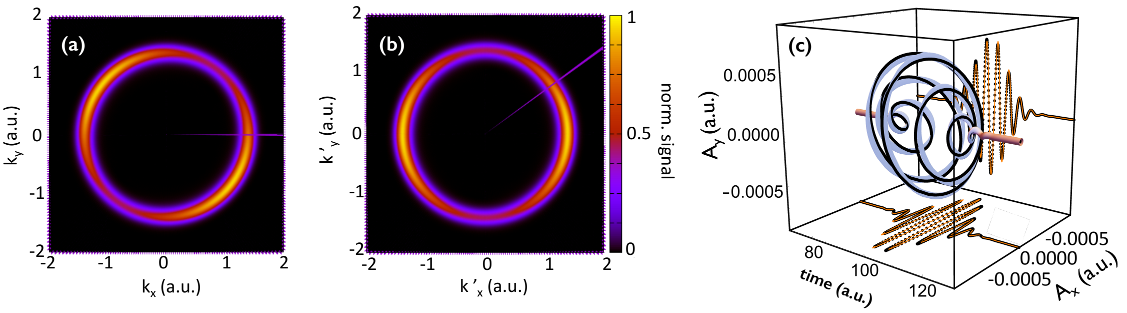

Supplementary Figure 4: **Reconstruction of pulse with non-orthogonal components**. (a) Angularly-resolved XUV-only photoelectron spectra for the pulse in Eq. (43). (b) Same photoelectron distribution as in panel (a) but with an $\alpha = 36.2°$ rotation of the axes. (c) Pulse in Eq. (43) (orange are 2D projections) and its reconstruction (black line in 3D and black dots in 2D). The reconstruction is shown in the reference frame of panel (a).

Time-dependent phase

Now let us consider the time-dependent phase, $\varphi(t)$. Without the loss of generality, we set the $\varphi(t_0) = 0$, since we already know that this can be accomplished by simply

rotating the reference frame.

First, we note that time-dependence associated with the phase $\varphi(t)$ can be obtained from the standard FROG-CRAB spectrograms, measured separately for both x and y directions. This time dependence results in frequency shifts and chirps. It is only the degree of coherence and the degree of de-polarization between the two components that are missing in the FROG-CRAB measurement. In other words, in general there could still be a component, with the weight $\sin^2\gamma$, in which $\varphi(t)$ is random from shot to shot. This de-polarized component of the light can have any polarization between x and y with equal probability. To obtain $\sin^2\gamma$, we need an additional observable – this is the left-right asymmetry.

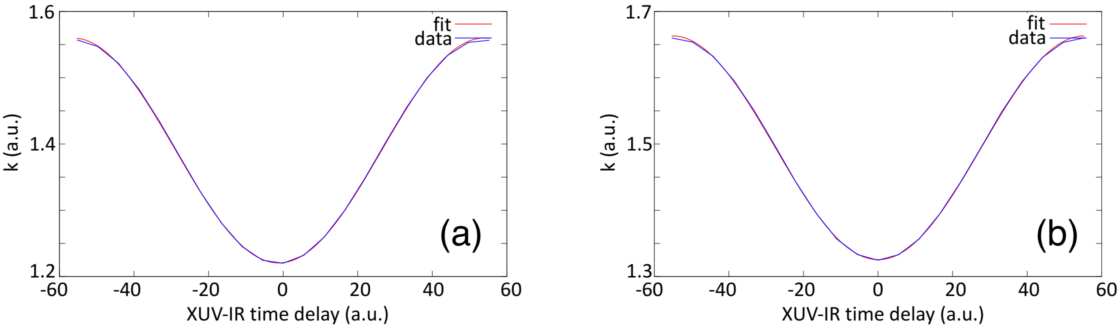

Supplementary Figure 5: **Streak traces of pulse with time-dependent phase between components**. Fit to the streaking spectrograms along the (a) $k = k_x$ axis and along the (b) $k = k_y$ axis. For (a), the fit yields $\omega_{x,\mathrm{XUV}} = 1.48$ a.u., for (b) the fit yields $\omega_{y,\mathrm{XUV}} = 1.63$ a.u..

We take the same pulse as in Fig.2b, with time-dependent ellipticity, but we now add the time-dependent phase $\varphi(t) = 0.15(t - t_{0\mathrm{XUV}})$. This corresponds to the frequency shift for the y component. From the fits to the centers of the FROG-CRAB spectrograms taken separately for x and y components, see Supplementary Figure 5, we identify the two different carrier frequencies for the x and y components. The frequency shift is accurately retrieved to be 0.15 a.u., that is about 4.1 eV.

We now measure the asymmetry. We align the IR polarization parallel to the major axis of the time-averaged ellipse and measure the spectra in the perpendicular direction, that is, at 90 and 270 degrees, see Supplementary Figure 6a for $\cos^2\gamma = 1$. The asymmetry in Supplementary Figure 6b is computed for the case of no depolarization. It is markedly different from the case of time-independent phase φ, that is it is also sensitive to $\varphi(t)$. Crucially, the asymmetry is very sensitive to $\sin^2\gamma$, see Supplementary Figure 6c,d computed for $\sin^2\gamma = 0.3$. Hence, the degree of coherence (or, alternatively, the de-polarization) can be easily extracted by fitting the asymmetry obtained from the theoretical calculations for different values of $\sin^2\gamma$ to the experimentally measured asymmetry. We also point out that one can take advantage of the great redundancy of this technique, by measuring the photo-electron signal along several angles and for several time delays. The magnitude of the asymmetry can also be magnified by increasing the IR field.

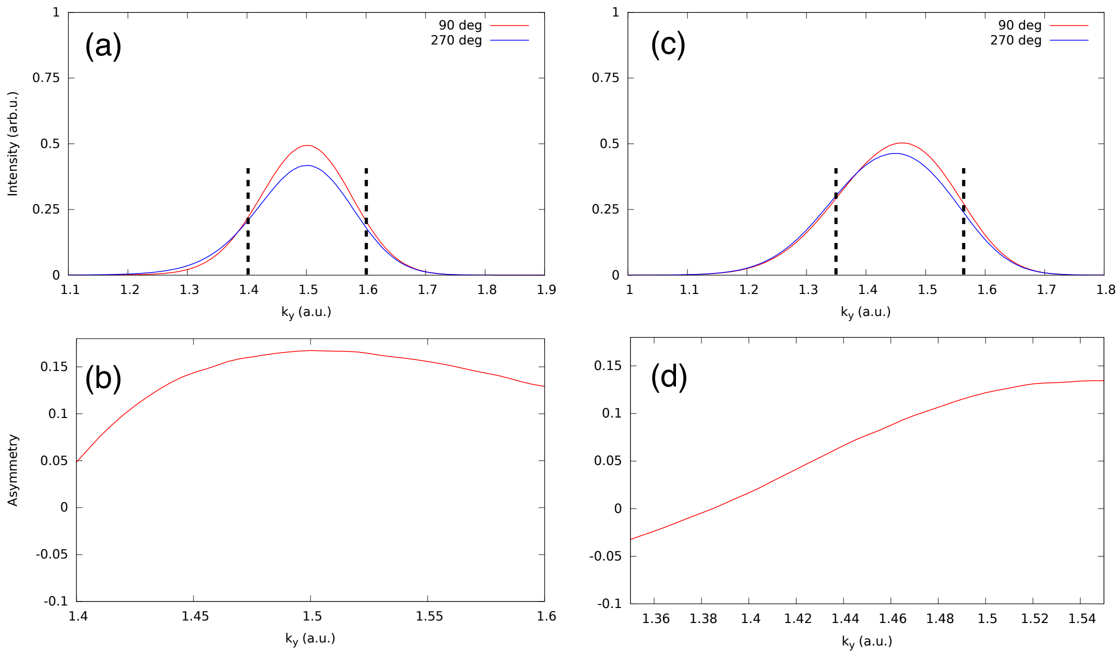

Supplementary Figure 6: **Asymmtries of pulses with time-dependent phase between components**. Photoelectron distributions at 90° and 270° and their asymmetry as a function of electron momentum. The IR field is polarized along the major axis of the XUV ellipse (along 0°) and the dashed lines indicate the region where we compute the asymmetry. (a,b) Perfectly polarized light, (c,d) partially polarized light ($\cos^2 \gamma = 0.7$). The asymmetry strongly depends on the degree of polarization.

Higher order terms (quadratic time dependence, etc.) are no different than first order terms; the chirp of the x and y components individually can be obtained by using FROG-CRAB, whilst the degree of coherence can be retrieved with the fitting procedure described above.

Supplementary Note 6: Amplitude fluctuation between x and y directions of polarized component

Here we consider random fluctuations of the amplitude along one specific direction and not the other, that is, fluctuating ellipticity from shot to shot. We have simulated the case in which the y component fluctuated from shot to shot, so that the ellipticity fluctuated from 0.7 to 0.8. We incoherently summed the spectra computed for ellipticity of 0.7, 0.725, 0.75, 0.775 and 0.8 (see Supplementary Figure 7). From the measurement of the asymmetry, we retrieved the ellipticity of $\epsilon = 0.74$, which is essentially the average of the fluctuations (see Supplementary Figure 8).

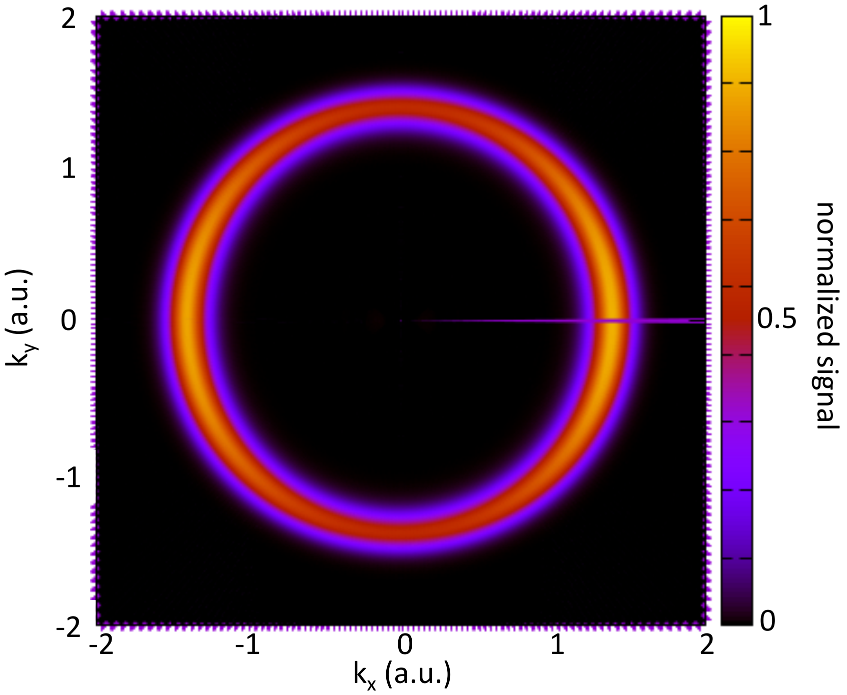

Supplementary Figure 7: **Spectra with amplitude fluctuation between components**. Angularly-resolved XUV+IR photoelectron spectra of averaged ellipticities (0.7, 0.725, 0.75, 0.775, 0.8) mimicking an experimental spectra with a fluctuation of the y component from pulse to pulse.

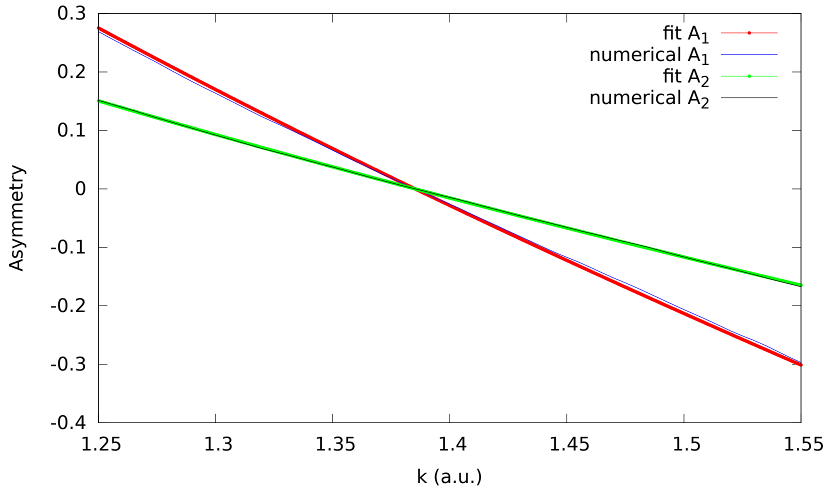

Supplementary Figure 8: **Asymmetry extraction with amplitude fluctuation between components**. Fitting of the asymmetries A_1 and A_2 for several measurements of a pulse with a fluctuating ellipticity (from 0.7 to 0.8).

Supplementary References

[1] L. V. Keldysh. Ionization in the field of a strong electromagnetic wave. *JETP*, **20** (5):1307–1314 (1965).

[2] F. Faisal. Multiple absorption of laser photons by atoms. *J. Phys. B: At. Mol. Opt. Phys.*, **6**:L89 (1973).

[3] H. R. Reiss. Effect of an intense electromagnetic field on a weakly bound system. *Phys. Rev. A*, **22**(5):1786–1813 (1980).

[4] J. Itatani, F. Quéré, G. L. Yudin, M. Yu. Ivanov, F. Krausz, and P. B. Corkum. Attosecond streak camera. *Phys. Rev. Lett.*, **88** (17):173903– (2002).

[5] K. Veyrinas, V. Gruson, S. J. Weber, L. Barreau, T. Ruchon, J. F. Hergott, J. C. Houver, R. R. Lucchese, P. Salieres, and D. Dowek. Molecular frame photoemission by a comb of elliptical high-order harmonics: a sensitive probe of both photodynamics and harmonic complete polarization state. *Faraday Discuss. Chem. Soc.*, **194** (0):161–183 (2016).

[6] Y. Mairesse and F. Quéré. Frequency-resolved optical gating for complete reconstruction of attosecond bursts. *Phys. Rev. A*, **71** (1):011401– (2005).

[7] Misha Ivanov and Olga Smirnova. How accurate is the attosecond streak camera? *Phys. Rev. Lett.*, **107** (21):213605– (2011).
